# Supplementary material for: Actin Branching Regulates Cell Spreading and Force on Talin, but not Activation of YAP
Source: Cell Mol Bioeng. 2025 Aug 4;18(3-4):271–82. doi: 10.1007/s12195-025-00852-3 (PMC12436249; doi:10.1007/s12195-025-00852-3)
Supplement: Supplementary file 1 — Supplementary file1 (DOCX 935 kb) [file 12195_2025_852_MOESM1_ESM.docx]

**Supplemental Figures**


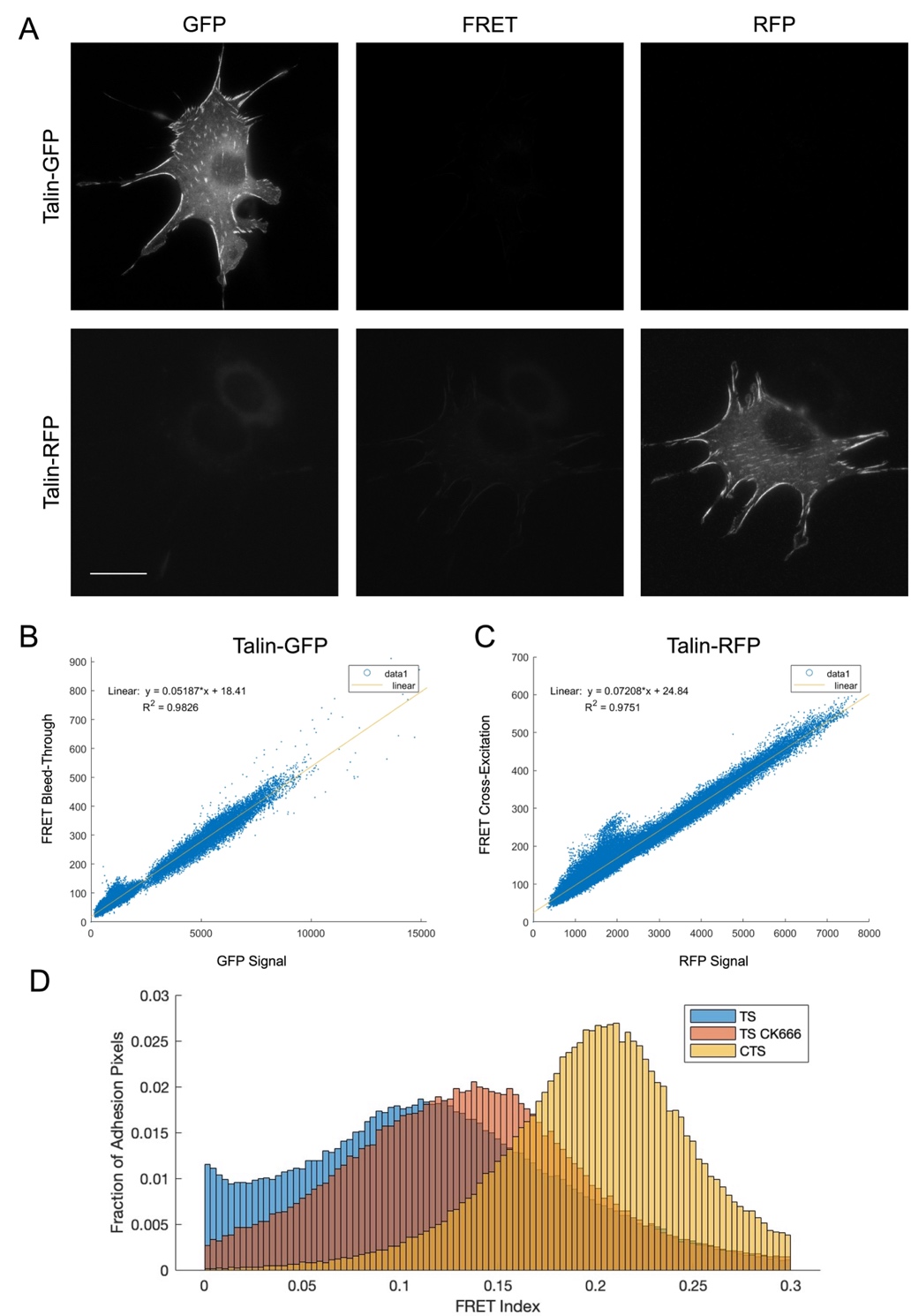


**Supplemental Figure 1:** Calculation of the bleed through and cross-excitation coefficients for FRET tension sensor imaging using a GFP tagged Talin (Talin-GFP) and an RFP tagged Talin (Talin-RFP). Example images of each construct expressed in 3T3 cells with each of the 3 channels captured for 3-image FRET calculations (A). Plots of GFP bleed through (B) and RFP cross-excitation (C) with linear fits to extract the bleed through and cross-excitation coefficients for the specific microscope settings used. Scale bar = 20μm. Histogram of pixel-wise data for FRET tension sensor in 3T3 fibroblast adhesions with or without CK666 compared to CTS control (D).


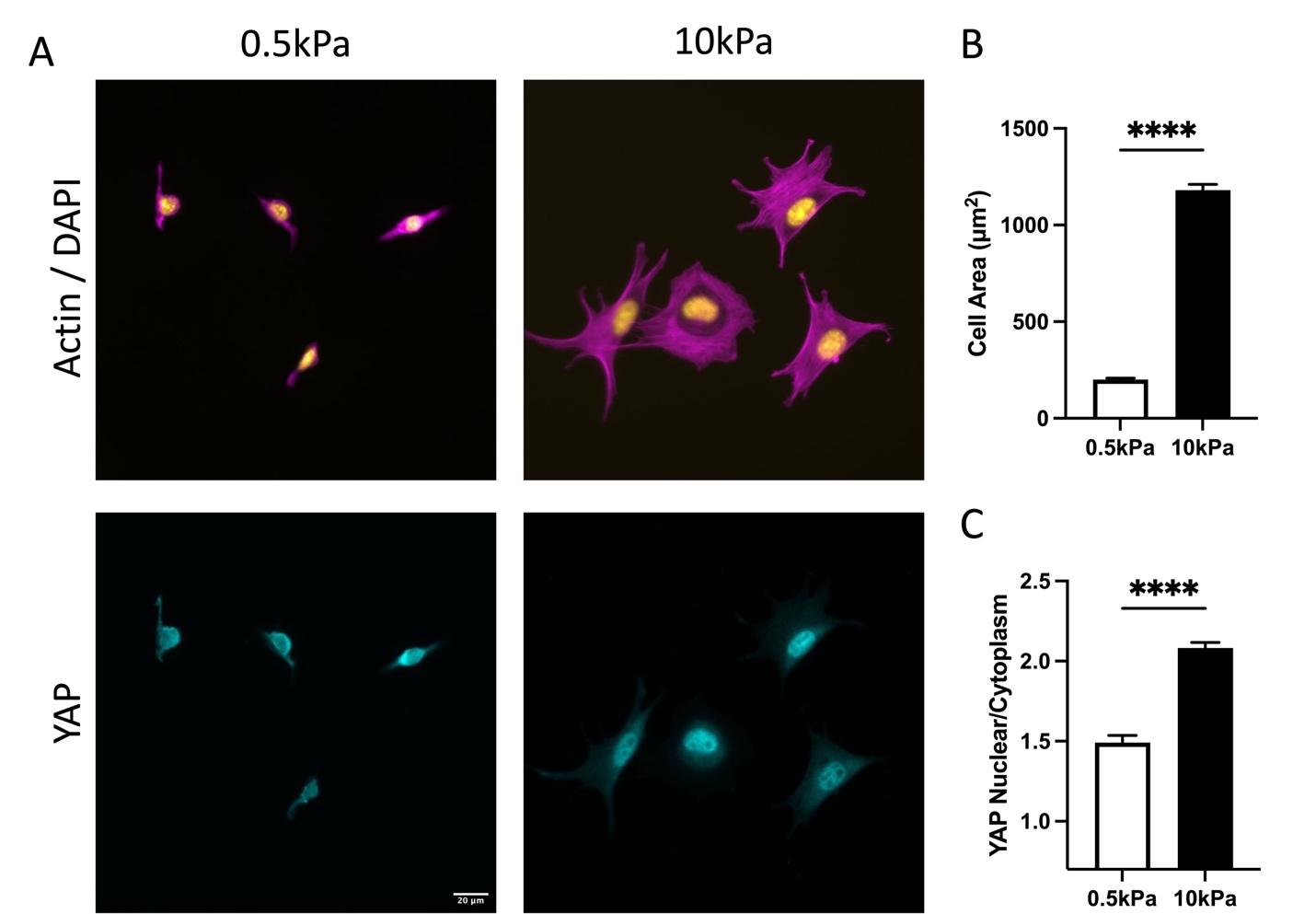


**Supplemental Figure 2:** Low stiffness control using a 0.5kPA polyacrylamide gel coated with fibronectin and compared to the 10kPA PDMS gel used for TFM and other deformable substrate experiments. (A) Representative images of Actin/DAPI (magenta/yellow) and YAP (cyan). Quantification of cell area (B) and nuclear to cytoplasmic ratio of YAP (C). Mean +/- SEM, two-sided t-test, **** p<0.0001, n=65-190 cells per group. Scale bar = 20μm.

**
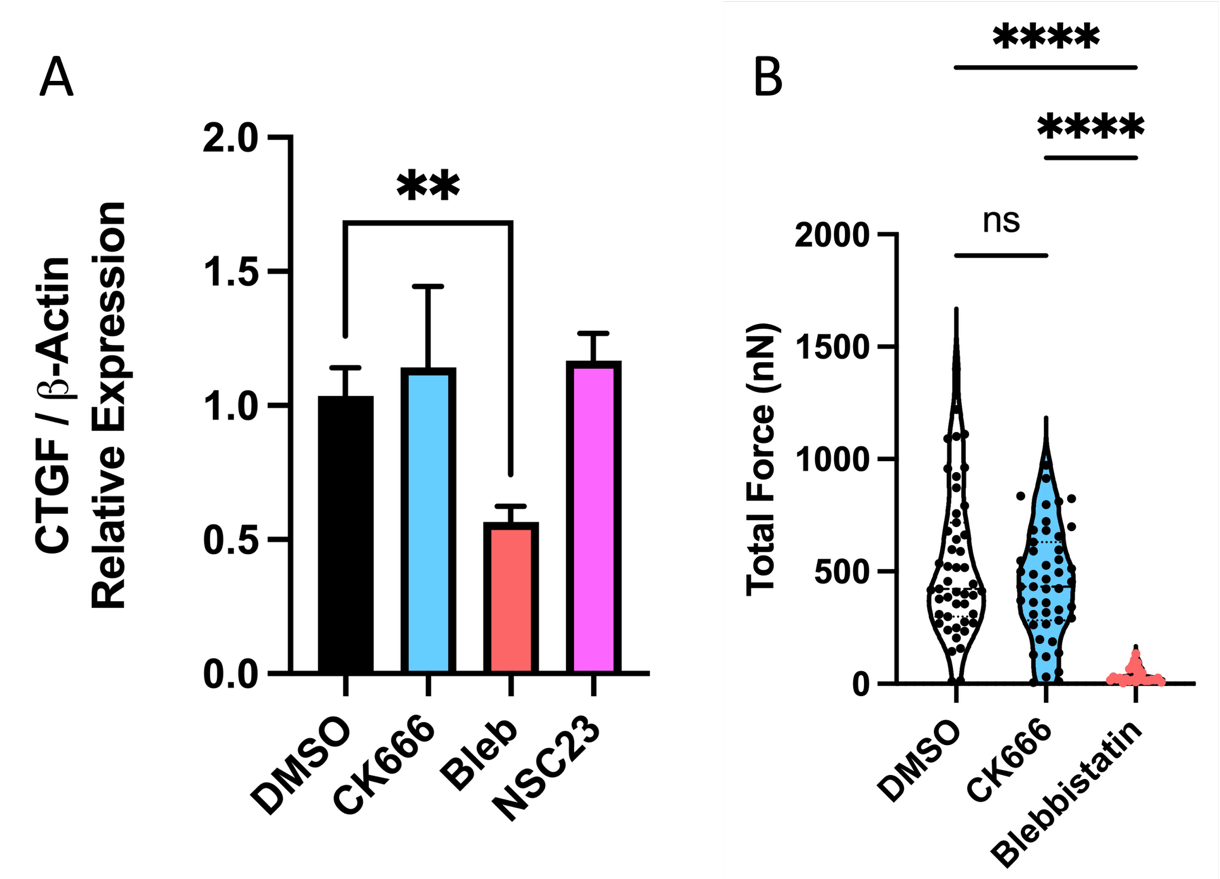
**

**Supplemental Figure 3:** (A) qPCR for expression of the YAP target gene CTGF normalized to expression of β-Actin for cells treated with inhibitors for 24 hours on fibronectin coated glass. DMSO (no inhibitor control), inhibition of Arp2/3 (CK666 50μM), inhibition of myosin (Bleb 10 μM) or inhibition of Rac (NSC23 50μM). Mean +/- SEM, n=3-8 samples per group from 3 independent experiments. One-way ANOVA with Bonferroni’s Post Hoc, ** p<0.01. (B) Total force per cell for traction force experiments in Figure 5A-B (on Fibronectin coated 10kPa PDMS with or without inhibitors). Violin plot indicates distribution, mean, and quartiles. Non-parametric Kruskal-Wallis test with Dunn’s multiple comparison test. n=22-44 cells per group from 3 independent experiments. **** p<0.0001.


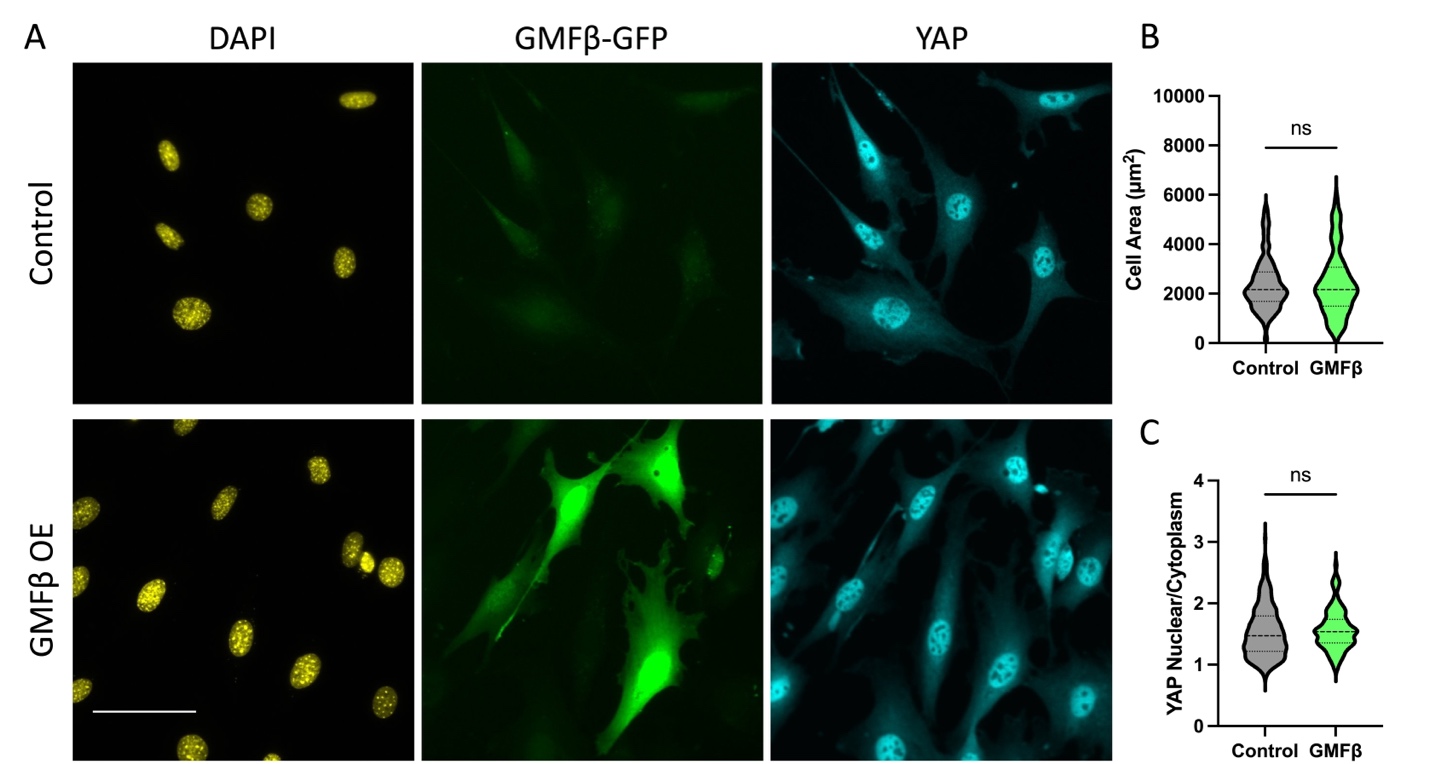


**Supplemental Figure 4:** Over expression of the actin debranching protein GMFβ-GFP in 3T3 cells seeded for 24 hours on fibronectin coated glass, with representative images (A) of nucleus (DAPI, yellow), GMFβ-GFP (green), and YAP (cyan). Quantification of cell spread area (B) and YAP nuclear to cytoplasmic ratio (C). Violin plots indicate distribution, mean, and quartiles, n>72 cells per group. Scale bar = 50μm.


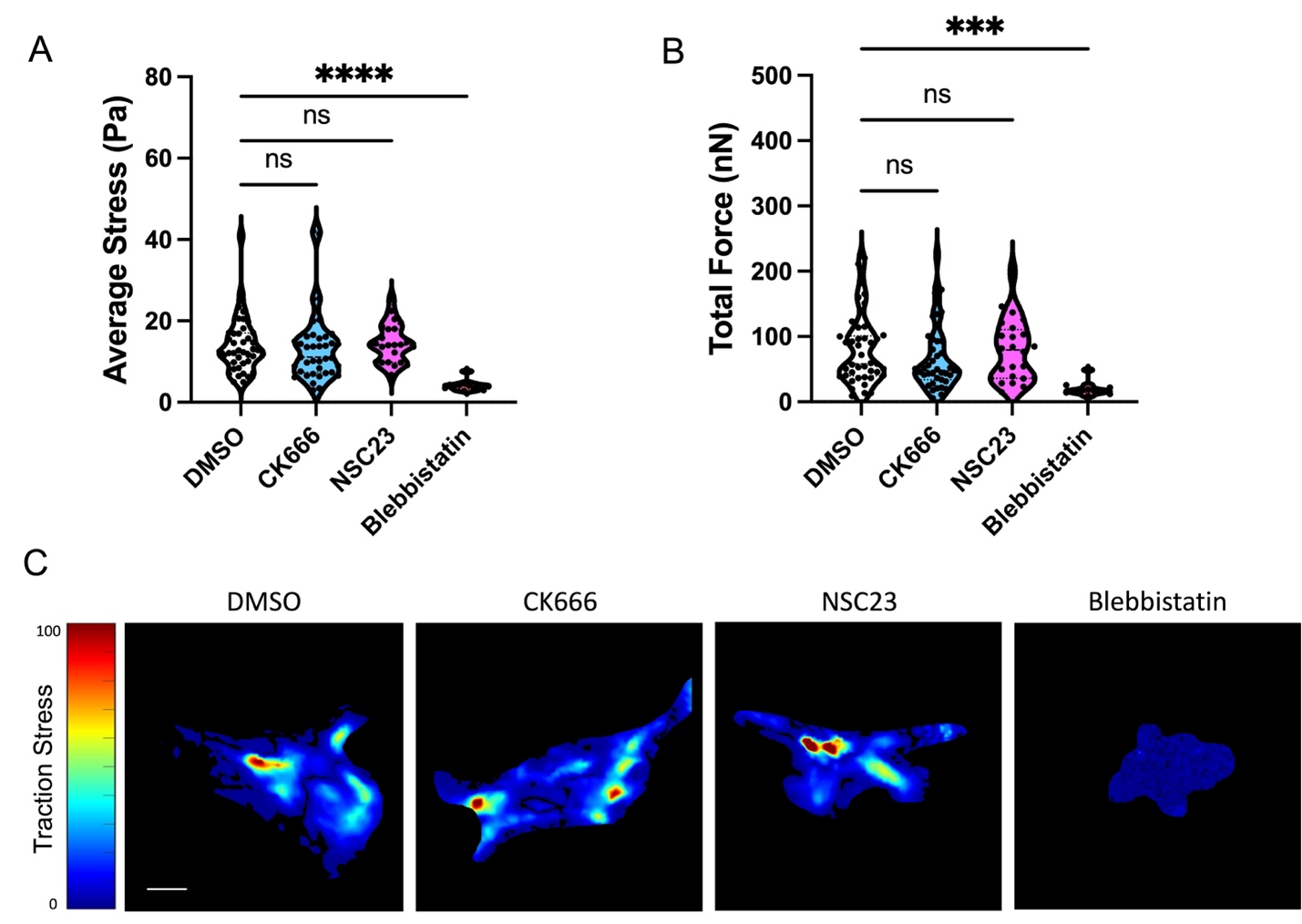


**Supplemental Figure 5:** Traction force microscopy on 2kPA PDMS gels coated with fibronectin for 3T3 cells treated with Arp2/3 inhibitor (CK666, 50μM), Rac inhibitor (NSC23, 50μM), or myosin inhibitor (Blebbistatin, 10μM), compared to DMSO control. Quantification of average traction stress per cell (A), total force per cell (B), and representative heat maps of traction forces (C). Violin plots indicate mean and quartiles, n=12-41 cells per group from 2 independent experiments. Scale bar = 10μm.
